# Structure–Function Relationships and Health-Promoting Properties of the Main Nutraceuticals of the Cactus Pear (*Opuntia* spp.) Cladodes: A Review

**DOI:** 10.3390/molecules29194732

**Published:** 2024-10-07

**Authors:** Meriyem Koufan, Basma Choukrane, Mouaad Amine Mazri

**Affiliations:** 1 Natural Resources and Local Products Research Unit, Regional Center of Agricultural Research of Agadir, National Institute of Agricultural Research, Avenue Ennasr, BP 415 Rabat Principale, Rabat 10090, Morocco; 2 Plant Breeding and Quality Research Unit, Regional Center of Agricultural Research of Marrakech, National Institute of Agricultural Research, Avenue Ennasr, BP 415 Rabat Principale, Rabat 10090, Morocco; basma.choukrane@inra.ma; 3 Agro-Biotechnology Research Unit, Regional Center of Agricultural Research of Marrakech, National Institute of Agricultural Research, Avenue Ennasr, BP 415 Rabat Principale, Rabat 10090, Morocco

**Keywords:** bioactive compounds, biological activity, cactus cladode, *Opuntia* spp., nutraceuticals, health-promoting properties

## Abstract

Over the past decade, several studies have established a direct link between functional foods, nutraceuticals, and a reduced risk of oxidative-stress-related diseases. Nutraceuticals, which encompass a variety of bioactive molecules, exhibit both nutritional and therapeutic properties. The cactus pear (*Opuntia* spp.) is a plant genus with many species recognized as functional foods, largely attributed to their high content of nutraceuticals, including polyphenols, fatty acids, vitamins, amino acids, pigments, and phytosterols. These compounds of different structures and functions possess different biological activities, contributing to the health-promoting properties of cactus pear. This makes cactus pears a valuable plant for the food, cosmetic, and pharmaceutical industries. While extensive research has focused on the nutritional profile of cactus pear fruits, the cladodes have received comparatively limited attention. Notably, the nutritional composition of cladodes can exhibit considerable variability, influenced by species and growing conditions. Furthermore, although various bioactive compounds have been identified in cladodes, studies elucidating their mechanisms of action, health benefits, and potential therapeutic applications remain insufficient. Addressing these gaps is crucial for enhancing the understanding and utilization of cactus pear cladodes. This paper provides a comprehensive overview of the structure–function relationships of the main nutraceuticals found in cactus pear cladodes. It synthesizes data from recent and relevant literature to elucidate the content of these compounds in relation to species and geographical origin, while also detailing the main biological activities and health-promoting benefits associated with cactus pear cladodes.

## 1. Introduction

The cactus pear (*Opuntia* spp.) is a genus within the family Cactaceae [1]. It is native to Mexico and comprises more than 300 species, with *O. ficus indica* being the most economically important [2]. Today, the cactus pear is cultivated across various countries and regions worldwide [3]. This genus is cultivated for multiple purposes, including the consumption of fruit and fruit-derived products, as well as applications in pharmaceuticals, nutraceuticals, and cosmetics. Additionally, the cactus pear plays important ecological roles, such as biodiversity preservation and environment protection, as well as being used as forage. Thus, it contributes significantly to livelihood security and economic support [4].

Bioactive compounds are natural substances with diverse biological activities [5]. They can be classified as either essential or non-essential and may also be generated during food processing, offering substantial health benefits [6,7]. They play crucial roles in human growth and development, contributing significantly to overall well-being through various beneficial effects, including disease prevention, antiaging, energy provision, and the maintenance of physiological functions [5,8]. Bioactive compounds can be obtained from different sources, including plants, fungi, and bacteria [9]. Plants rich in bioactive compounds are often categorized as either medicinal or poisonous [10].

In recent years, many studies have been conducted to profile the compound content of cactus pears [11,12,13]. These investigations have confirmed that cactus pear plants are a rich source of bioactive constituents, including polyphenols, fatty acids, vitamins, amino acids, natural pigments, and phytosterols. These compounds are essential for enhancing human health and preventing diseases [14]. Consequently, the cactus pear represents a genus with significant potential in both the food and biomedical industries.

Due to its richness in bioactive compounds, the various biological activities of cactus pears have been investigated. Previous studies have shown that cactus pear fruits possess antioxidant, antimicrobial, anti-inflammatory, neuroprotective, blood-sugar-regulating, and antigenotoxic properties, among others [15]. Cactus pear seed oil has demonstrated potent antioxidant and antimicrobial activities [11]. On the other hand, cactus pear cladodes have been linked to the prevention of chronic diseases and exhibit wound-healing properties [16]. These biological effects can be attributed to the structure–function relationships and synergistic interactions of the bioactive compounds present in the plant.

To date, most research on the chemical composition and biological activities of cactus pear has predominantly focused on its fruits. This is mainly due to the fact that fruits are the main part used for human consumption, either fresh or processed into various products [4].

Cactus pear cladodes are rich in essential nutrients and bioactive compounds, which have significant health-promoting properties, making them a valuable food source. Understanding these compounds can facilitate the development of functional foods and nutraceuticals. The nutritional profile of cladodes complements that of the fruits, providing a different set of health benefits. Additionally, cladodes can be used in various culinary applications, as well as in the cosmetic and pharmaceutical industries. Investigating the properties of cladodes can increase their utilization and foster innovation in product development. Compared to fruits, cladodes have received limited attention in research [17,18]. Exploring their potential could uncover new applications and benefits that are currently overlooked, as well as provide a deeper understanding of their unique contributions to nutrition, agriculture, and industry, thereby expanding their use and functionalities. Besides, the contents of bioactive compounds in cactus pear cladodes may vary considerably depending on factors such as species, cultivar/genotype, and geographic origin. Along this line, measurements should account for these factors. Generally, *O. ficus indica* has been the most extensively studied cactus pear species [17,18,19,20,21]. Other species, such as *O. stricta*, *O. dillenii*, and *O. littoralis*, have also demonstrated a richness in bioactive compounds and potent biological activities [22,23,24].

This paper presents a comprehensive overview of the structure and functions of the most abundant bioactive compounds found in cactus pear cladodes. Additionally, it gathers the most relevant studies that examine the roles and amounts of these compounds, with a particular focus on the variations between cactus pear species and their geographical origin. The primary biological activities of cactus pear cladodes are also emphasized.

## 2. Structure–Function Relationships of the Major Nutraceuticals of Cactus Pear Cladodes

Cactus pear cladodes are rich in bioactive compounds, including polyphenols, fatty acids, vitamins, sterols, amino acids, and natural pigments [25]. These compounds confer to *Opuntia* cladodes disease-preventing properties. Many health-promoting properties such as antioxidant, anticancer, antimicrobial, anti-inflammatory, and antidiabetic effects have been scientifically demonstrated [26]. Cactus pear cladodes also serve as livestock feed in arid and semi-arid regions [4]. They are also used as a food additive and in the pharmaceutical industry [4]. In some countries, cladodes are consumed fresh in salads or processed into various products [26]. The biological activities of cactus pear cladodes can be attributed, in part, to the structure–function relationship of their bioactive compounds.

### 2.1. Polyphenols

Polyphenols are a major group of secondary metabolites and bioactive compounds found in fruits and vegetables. These compounds have a polyphenol structure, characterized by aromatic rings containing one or more hydroxyl groups [27,28,29]. The polyphenol class also includes molecules with a single phenol ring and encompasses over 8000 distinct phenolic structures [27,29,30]. The mechanisms by which polyphenols exert their effects are closely linked to their chemical structure. These mechanisms may involve the inhibition of enzymes, chelation of trace metal ions essential for the free radical production, scavenging of reactive free radicals, and the regeneration of membrane-bound antioxidants [29]. By modulating cellular responses to oxidative stress—an underlying factor in many diseases—polyphenols can mitigate harmful effects [28]. This modulation influences cell signaling pathways, further contributing to their protective effects [28].

Polyphenols were reported to have a potent antioxidant activity, which confers various health-promoting benefits, including anticancer, anti-inflammatory, antiaging, and antidiabetic properties [28,31]. They are also recognized for their protective effects against chronic diseases [28]. According to Belščak-Cvitanović et al. [29], the strong antioxidant properties of polyphenols stem from their ability to neutralize free radicals by providing an electron or hydrogen atom, depending on the degree of methoxylation and the number of hydroxyl groups.

Polyphenols have a wide range of industrial applications. In the food and beverage sector, they act as effective preservatives, extending shelf life and preventing spoilage, while also being incorporated into functional foods that provide additional health benefits. In the pharmaceutical industry, polyphenols are formulated into capsules and powders to reduce inflammation and support cardiovascular health. Additionally, the cosmetic industry uses their potent antioxidant effects in skincare products aimed at combating skin aging and oxidative stress [32,33,34].

Among all polyphenols, flavonoids represent the largest and most extensively-studied group [27,35]. Other polyphenol groups include phenolic acids, stilbenes, and lignans. This classification is based on the number of phenol rings and the structural elements that connect them [36]. The content of polyphenols in plants varies significantly among species, genotypes, plant materials, age, and growing conditions [27].

Polyphenols are major constituents of cactus pear cladodes. However, their compositions and concentrations can vary widely among species, cultivars/genotypes, plant material, and the geographic origin. Sánchez et al. [37] compared the total phenols and total flavonoids in fully grown cladodes from seven cultivars of *O. ficus indica* and one cultivar of *O. streptacantha* from Mexico. The study revealed significant differences among the eight cactus pear cultivars evaluated. The total phenolic content ranged from 1.49 mg GAE/g DW in *O. ficus indica* cv. Forrajero Mina to 4.27 mg GAE/g DW in *O. ficus indica* cv. Copena F1, while the total flavonoid content ranged from 15.4 mg QE/g DW in *O. ficus indica* cv. Real de Catorce to 36.6 mg QE/g DW in *O. ficus indica* cv. Jalpa.

In fresh cladodes of *O. ficus indica* from South Africa, the total phenolic content ranged from 239.47 mg/kg in cv. Ofer to 270.93 mg/kg in cv. Gymno-Carpo; whereas, *O. robusta* exhibited a phenolic content of 42.84 mg/kg [38]. Cladodes of *O. ficus indica* from Egypt had a total phenolic content of 119.66 mg/100 g [18], while those from Jeju Island (Republic of Korea) showed a total polyphenol content of 1.85 mg/g and a total flavonoid content of 1.29 mg/g [39].

In *O. ficus indica* from Alicante (Spain), significant differences in phenolic content were observed based on cladode age and cultivar [40]. In young cladodes (less than one year old), the total phenolic content ranged from 5.3 to 14.3 mg/g DW, while in older cladodes (two years old), it varied from 4.2 to 12.4 mg/g DW. The phenolic composition revealed 26 compounds in the young cladodes and 25 in the older ones, with significant differences among cultivars. Flavonoids, particularly flavonols, were identified as the predominant phenolic group in the cladodes [40].

Haile et al. [41] evaluated total polyphenols, flavonoids, and tannins in both spiny and spineless *O. ficus indica* from various regions of Ethiopia. They found significant differences based on the cladode type and the sampling area. The total polyphenol content ranged from 18 to 47.5 mg GAE/g DW in spineless cladodes and from 23 to 71.4 mg GAE/g DW in spiny cladodes. The flavonoid content ranged from 6.44 to 13.1 mg CE/g DW in the spineless cladodes and from 7.29 to 25 mg CE/g DW in the spiny ones. The total tannin content ranged from 9 to 25.6 mg GAE/g DW in spineless cladodes and from 9.1 to 41.2 mg GAE/g DW in spiny cladodes.

### 2.2. Fatty Acids

Fatty acids are bioactive compounds highly valued for their biological properties [42]. They are aliphatic monocarboxylic acids with different structures and functions, categorized as short-, medium-, or long-chain fatty acids, depending on the number of atoms in the aliphatic chain [43,44]. The carbon chain of fatty acids can contain between 2 and 36 carbon atoms [45]. Besides, fatty acids can be either saturated or unsaturated, depending on the presence of a double bond in their molecular structure, which confers to their biological properties [46,47]. Based on the number of double bonds, the unsaturated fatty acids can be further classified into monounsaturated or polyunsaturated [47]. In addition, fatty acids are also categorized as essentials or non-essentials [46]. Essential fatty acids cannot be synthesized by the human body; although, they are involved in regulating various bodily functions [46]. Thus, they are obtained through diet.

Fatty acids are known to regulate inter- and intracellular signaling molecules and exhibit anticancer activities [43]. They are essential for human development, influencing gene expression, and cellular communication processes [45]. Saturated fatty acids provide energy, maintain membrane fluidity, and regulate gene transcription [47,48]. However, an excessive intake of saturated fatty acids has been linked to health issues such as atherosclerosis and coronary heart disease [49]. Polyunsaturated fatty acids have been associated with many numerous health benefits, including antifungal properties and the potential to inhibit carcinogenesis and the development of atherosclerosis [42,50].

The diverse chemical properties of fatty acids lend them a wide range of industrial applications. In the food industry, they are used as emulsifiers, stabilizers, and preservatives, while also being incorporated into nutritional supplements. In the cosmetic and pharmaceutical industries, fatty acids are used as emollients and surfactants, enhancing product texture and effectiveness. Fatty acids are also valued for their potential therapeutic properties [51,52,53].

The fatty acid composition of cactus pear cladodes has been evaluated in different species from different geographic locations. According to many authors, oleic, linoleic, and palmitic acids are the predominant fatty acids found in cactus pears.

In the cladodes of *O. ficus indica* from Egypt, unsaturated fatty acids accounted for 69.23% of the total fatty acid content, while saturated fatty acids made up 30.77%. Among these, linoleic acid (24.81%) was the dominant polyunsaturated fatty acid, while oleic (10.39%) and palmitic (7.53%) acids were the most abundant monounsaturated and saturated fatty acids, respectively [18]. In *O. ficus indica* cladodes from Spain, the fatty acid composition varied significantly among cultivars, with high percentages of linoleic, linolenic, oleic, and palmitic acids present in all cultivars [54].

The cladodes of *O. humifusa* from Korea exhibited a high content of polyunsaturated fatty acids (62.13%), with linoleic acid being the major compound at 38.88%. The content in monounsaturated fatty acids was 13.93% (8.42% oleic acid), while saturated fatty acids constituted 23.94% (16.73% palmitic acid) [55]. In *O. Sulphurea* from Argentina, Carreira et al. [56] reported that fatty acids are a major constituent of the cladodes, with linolenic acid being the major compound. In *O. microdasys* and *O. macrorhiza* from Tunisia, saturated fatty acids represented 61% and 56% of the total fatty acids, respectively. The percentages of monounsaturated fatty acids were 6.7% and 7.8%, while those of polyunsaturated fatty acids were 33% and 36%, respectively. The main fatty acids detected in both species were palmitic (18–20%) and linoleic acids (20–24%) [57].

### 2.3. Vitamins

Vitamins are organic molecules generally obtained from the diet and are essential for human health and proper functioning. Thirteen vitamins are necessary for optimal health: A, D, E, K, C, and the B vitamins (B1, B2, B3, B5, B6, B7, B9, and B12) [58,59]. These vitamins are classified into two groups depending on their solubility: fat-soluble and water-soluble [58,59,60]. The fat-soluble vitamins—A, D, E, and K—are known for their potent antioxidant properties [59,60,61]. Water-soluble vitamins, such as C and the B-complex vitamins, cannot be synthetized by the body [59,60,61,62].

While vitamins vary significantly in structure, even those within the B-complex group, they serve critical roles and are indispensable for cell function, growth, and development [58,60,61,63]. Vitamins can be a single compound or a large group of compounds [63]. The structurally and functionally unrelated vitamins are essential for human health and functioning [60,61]. Their functions can be specific or broad-ranging, making them essential for overall health [58,59,60,61,64]. Vitamins are integral to various biochemical and physiological processes, including cell and brain function, human metabolism, and cellular regulation [59,61]. Both deficiencies and excesses of vitamins can lead to health issues [59].

Due to their importance, vitamins are widely used in the food, cosmetic, and pharmaceutical industries. In the food sector, they are often added to processed foods and beverages to enhance nutritional value, and dietary supplements are developed to promote health and prevent deficiencies. In pharmaceuticals, vitamins play a crucial role in formulating medications to address specific health conditions, and they are frequently used in clinical settings for rapid absorption and treatment. In the cosmetic industry, vitamins are incorporated into various products for their powerful antioxidant properties, contributing to skin health and antiaging effects [65,66,67].

Among all vitamins, C and E (i.e., ascorbic acid and tocopherol) have been the most extensively studied in cactus pears. In *O. ficus indica* from Jeju Island (Republic of Korea), the vitamin C content was measured at 71.2 mg% [39]. In the cladodes of *O. humifusa* from Korea, vitamins A and C were reported to be 3.28 and 17.33 mg%, respectively [55]. In Mexican *O. ficus indica* cvs. Milpa Alta and Atlixco, the total vitamin C content was 63.92 and 57.03 mg/100 g DW, with ascorbic acid levels of 19.21 and 25.52 mg/100 g DW, respectively [68]. In *O. ficus indica* from Egypt, the contents of vitamins C and E were 15.94 mg/100 g and 1.37 μg/100 g, respectively, while vitamin D was not detected [18].

Du Toit et al. [38] compared ascorbic acid contents in the fresh cladodes of four *O. ficus indica* cultivars (Gymno-Carpo, Meyers, Nepgen, and Ofer) and *O. robusta* from South Africa. In *O. ficus indica* cultivars, the ascorbic acid content ranged from 26.78 to 77.40 mg/100 g, compared to 24.04 mg/100 g in *O. robusta*. In Tunisian *O. macrorhiza* and *O. microdasys*, ascorbic acid contents were 0.017 and 0.0061 g/100 g DW, respectively. The total tocopherol content was 5.1 mg/100 g DW for *O. macrorhiza* and 6.9 mg/100 g DW for *O. microdasys*, with α-tocopherol being the primary form, found at 4.9 mg/100 g DW in *O. macrorhiza* and 5.3 mg/100 g DW in *O. microdasys*. δ-tocopherol was not detected in *O. macrorhiza* [57]. Lanuzza et al. [69] assessed tocopherol levels in young Sicilian *O. ficus indica* cladodes, finding α-tocopherol at 98.4 mg/kg, β-tocopherol at 18.3 mg/kg, and ɣ-tocopherol at 2.3 mg/kg, while δ-tocopherol was not detected.

### 2.4. Amino Acids

Amino acids are bioactive organic macromolecules and fundamental components of proteins [70,71]. In plants, they are synthesized in the roots and transported to other parts via xylem sap [72]. Amino acids play crucial roles in plant development, serving as precursors for protein synthesis and energy sources [73,74]. Amino acids consist of an amino group, a carboxylic acid group, a central alpha-carbon atom, and a side chain [70,73]. They vary in electric charge, size, polarity, structure, hydrogen-bonding capacity, and chemical reactivity [75,76]. Some amino acids contain two carboxyl or two amino groups, while others have hydroxyl or sulfhydryl groups in their side chain [77]. The dehydration reaction between the carboxyl group of one amino acid and the amino group of another creates a peptide bond that leads them to join each other [75].

Amino acids are involved in numerous biochemical processes, including protein synthesis, lipogenesis, glucose generation, and energy production, and some serve as hormone precursors, contributing to body development [78,79]. The role played by amino acids in the different biochemical processes depends strongly on their structures, particularly the amino groups [78]. Additionally, the conformational stability of proteins relies on the interactions of the amino acids’ functional groups with water [70].

Amino acids have various biological and chemical applications, particularly in the food, cosmetic, and pharmaceutical industries, and as fertilizers [70,71,76]. They enhance the flavor and nutritional value of processed foods, serve as active ingredients or excipients in pharmaceuticals, and are essential for synthesizing therapeutic peptides and proteins. In cosmetics, amino acids are incorporated for their moisturizing and antiaging properties [80]. Based on their dietary importance, amino acids can be classified as essential or non-essential, with essential amino acids being transported to the brain via the blood [81,82].

The amino acid composition of cactus pears varies significantly by species and geographic origin, with glutamic acid generally being the predominant amino acid in *Opuntia* fruits and cladodes. Glutamic acid can be synthesized from other amino acids such as proline, arginine, and histidine, and it can also be converted into alpha-ketoglutarate, alanine, aspartate, or ammonium [83].

Research on amino acid content in cactus pear cladodes is limited. *O. ficus indica* from Jeju Island (Republic of Korea) has a total amino acid content of 6130.04 mg%, with glutamic acid as the major component at 1543.15 mg% (25.2%) [39]. Hernández-Urbiola et al. [84] evaluated the amino acid composition of *O. ficus indica* cv. ‘Redonda’ from Mexico at different growth stages, finding that glutamic acid (1.29–2.22 g/100 g of protein), phenylalanine (0.93–1.69 g/100 g of protein), and threonine (1.21–1.56 g/100 g of protein) were the most abundant amino acids across all stages (40 to 125 days). In *O. humifusa* from Yeosu, Korea, the total amino acid content was 4489.70 mg/100 g, with glutamic acid (609.90 mg/100 g), aspartic acid (531.81 mg/100 g), and leucine (412.54 mg/100 g) being the major compounds [55].

### 2.5. Natural Pigments

Natural pigments are bioactive compounds and chemically heterogeneous molecules with chromophore units that contain electron-conjugated systems [85,86]. They can be classified by their origin, utilization, and chromophore structure [86]. In plants, the primary classes of natural pigments include flavonoids, anthocyanin, carotenoids, and betalains [86], with the latter two being the most extensively studied in cactus pears.

Carotenoids are fat-soluble natural pigments known for their potent antioxidant activity [87,88]. While they are synthetized by plants, algae, and some microorganisms, humans cannot produce them, even though they play a crucial role in the human antioxidant defense system [88,89]. Accordingly, carotenoids must be obtained through the diet [88]. To date, more than 600 different carotenoids have been identified, with β-carotene being the most significant [89]. Most carotenoids share a 40-carbon backbone featuring conjugated double bonds and cyclic end groups [89]. The specific biological activities and functions of carotenoids are influenced by their specific structure, particularly the pattern and number of conjugated double bonds [89].

Betalains are water-soluble N-heterocyclic pigments that can be categorized into two structurally different classes: yellow–orange betaxanthins and red–violet betacyanins [90,91,92]. All betalains share betalamic acid as their chromophore, which serves as the core structure. They are further classified as betaxanthins or betacyanins based on the type of residue added to betalamic acid [90,91]. Betaxanthins contain various amino acids and amines condensed with betalamic acid; whereas, betacyanins feature a cyclo-3,4-dihydroxyphenylalanine (cyclo-Dopa) residue [90,93]. In cactus pears, the structures of betalains may vary depending on the species and genotype [94]. Due to their numerous health-promoting properties, including anti-inflammatory, anticancer, antihepatitis, antimicrobial, antimalarial, and antidiabetic effects, betalains are widely used in the pharmaceutical industry [95].

Natural pigments are widely used in the food industry as natural colorants and are also incorporated into dietary supplements due to their antioxidant activity and health-promoting properties [86,96,97,98]. In cosmetics, these pigments are valued for their vibrant colors and beneficial effects on skin, including UV protection. Additionally, they find applications in the pharmaceutical industry for their therapeutic properties, particularly their antioxidant and anti-inflammatory effects [86,99].

Only a few studies have evaluated the pigment content in the cladodes of cactus pears. In fresh cladodes from South African *Opuntia* species, the betacyanin content ranged from 3.17 mg/kg in *O. ficus indica* cvs. Gymno-Carpo and Ofer to 17.18 mg/kg in *O. robusta*. Betaxanthin levels varied from 2.22 mg/kg in *O. ficus indica* cv. Gymno-Carpo to 16.98 mg/kg in *O. ficus* indica cv. Ofer, while the carotene content ranged from 6.72 μg/g in *O. ficus indica* cv. Nepgen to 18.15 μg/g in *O. ficus indica* cv. Meyers [38].

Betancourt-Domínguez et al. [100] investigated total carotene and β-carotene contents of various sizes cladodes (small, medium, and large) from three cultivated *Opuntia* cultivars—Blanco sin Espinas, Blanco con Espinas, and Verde Valtierrilla—as well as a wild *Opuntia* plant from Mexico. They found that the β-carotene content varied significantly with genotype and cladode size, with the highest concentration (39.5 μg/g FW) in small cladodes of the wild plant and the lowest (14.5–16 μg/g FW) in cladodes of Blanco con Espinas. On the other hand, total carotene content did not show significant differences among the samples, ranging from 0.33 to 0.38 mg/g FW.

In cladodes of *O. ficus indica* from Mexico, Jaramillo-Flores et al. [101] reported a total carotenoid content of 231.8 μg/g on a dry basis, identifying three carotenoids: β-carotene (36%), cryptoxanthin (18%), and lutein (46%). Ramírez-Moreno et al. [68] reported total β-carotene contents of 61.32 mg/100 g DW in *O. ficus indica* cultivar Milpa Alta and 66.85 mg/100 g DW in cultivar Atlixco. The cladodes of *O. ficus indica* from Egypt exhibited a β-carotene content of 57.51 μg/100 g [18].

### 2.6. Phytosterols

Phytosterols, or plant sterols, are cholesterol-like molecules that naturally occur in plants. They exist in various forms, including free alcohols, fatty-acid esters, steryl glycosides, and acylated steryl glycosides [102,103,104,105]. More than 200 types of phytosterols have been identified in plants [103]. In edible oils, phytosterols are found in both free and esterified forms [104].

Structurally, phytosterols resemble cholesterol, featuring a four-ring steroid nucleus, 3β-hydroxyl group, and a double bond at carbon-5 [102,103,104,105]. They typically contain 28 or 29 carbon atoms and differ from cholesterol in the configuration of their side chains [103,104,106]. The side chains of the most abundant phytosterols have 9 to 10 carbon atoms; whereas, cholesterol’s side chain consists of only 8 carbon atoms [103,104]. Besides, phytosterols may contain an extra methyl or ethyl group or a double bond [103].

Phytosterols are associated with a wide range of biological functions, with their most notable effect being the potent reduction in cholesterol levels [103,106]. Studies have shown that phytosterols can lower serum low-density lipoprotein cholesterol concentrations, thereby helping to prevent cardiovascular diseases [103]. They achieve this by reducing the absorption of intestinal cholesterol, playing a crucial role in cholesterol elimination [102,105].

In addition to their cholesterol-lowering properties, phytosterols have been linked to a reduced risk of cancer by enhancing immune response recognition of cancer cells and preventing tumor progression and metastasis [105]. They also help stabilize phospholipid bilayers in cell membranes and regulate membrane metabolism, permeability, and fluidity [104,105].

Today, phytosterols are widely used in the food, cosmetic, and pharmaceutical industries. They are commonly added to functional foods to help lower cholesterol and promote heart health, and they are available as powdered supplements for cholesterol management. Additionally, phytosterols are incorporated into skincare products for their skin-conditioning properties and potential to improve skin barrier function, as well as for their anti-inflammatory effects [107].

Few studies have examined the phytosterol content of cactus pear cladodes. Most research has focused on the sterol composition of cactus pear seed oil, revealing that phytosterol profiles vary based on the geographic origin of the plant, but in all cases, with β-sitosterol consistently being the dominant phytosterol [108,109,110,111].

For cactus pear cladodes, it was reported that *O. ficus indica* contains a wide range of phytosterols, including β-sitosterol, β-campesterol, campestanol, Δ5-avenasterol, Δ7-stigmasterol, and stigmastanol. The content of these compounds varies with the maturity stage of the cladodes [112,113]. Similar to cactus seed oil, β-sitosterol has been identified as the predominant phytosterol in cactus pear cladodes [113].

## 3. Health-Promoting Properties of Cactus Pear Cladodes

**Figure 1:**
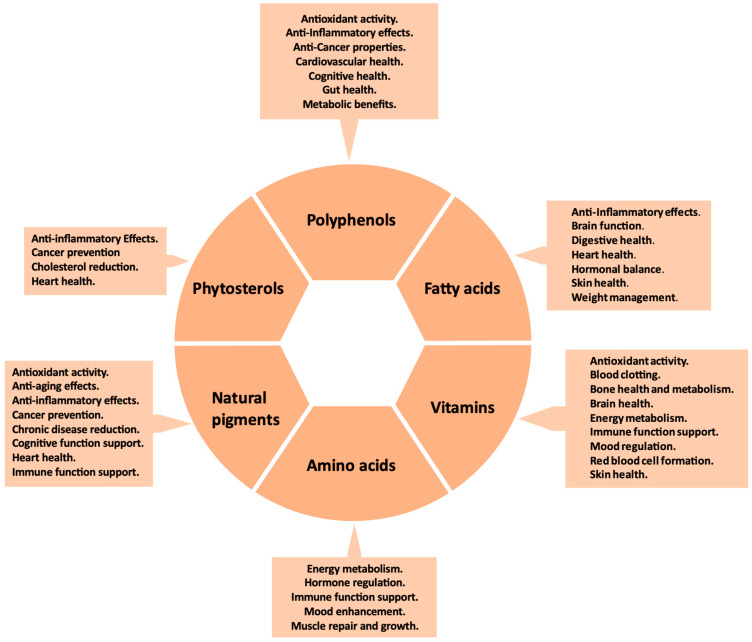
Health-promoting properties of polyphenols, fatty acids, vitamins, amino acids, pigments, and phytosterols.

In the past decade, numerous studies have demonstrated a direct relationship between functional foods and nutraceuticals and a reduced risk of oxidative-stress-related diseases [112]. Cactus pear cladodes have gained attention across various industries, including pharmaceuticals and cosmetics. This is due to their richness in nutraceuticals and bioactive compounds that promote human health and help prevent many diseases (Table 1; Figure 1). This has spurred research into their potential biological activities and health benefits.

Cactus pear cladodes are rich in fiber, minerals, and phenols [114]. Their polyphenol content contributes to their significant antioxidant activity [115], with notable polyphenols including rutin, ferulic acid, isorhamnetin derivatives, and betalain pigments [116]. This makes cactus pear cladodes effective as safe food additives and functional foods [117]. Additionally, their potent antioxidant properties lend them an antiulcer effect, enhancing their antiviral and antibacterial characteristics. Extracts from prickly pear cladodes have demonstrated significant antiviral activity against the *Cucumber mosaic* virus, and the isolated protein Opuntin B inhibits other viruses, including Tobamovirus and *Zucchini yellow mosaic* virus [118,119]. Spineless cactus pear cladodes also show antibacterial activity against *Bacillus subtilis*, *Staphylococcus aureus*, and *Escherichia coli* [117].

Cactus pear cladodes exhibit antidiabetic and antiobesity properties, attributed to their high levels of complex carbohydrates, ascorbic acid, carotenes, malaxinic acid, and other bioactive compounds [120]. Malaxinic acid is particularly noted for its role in antiobesity benefits [121]. Cactus pear cladodes have hypoglycemic effects and may enhance glycemic management by influencing glucose absorption and reducing its release into the bloodstream [122]. As a result, they can lower cholesterol, LDL, triglycerides, and fat accumulation, while improving lipid metabolism, immune function, and glycemic control [123].

It was also revealed that cactus pear plants possess anticarcinogenic properties, particularly with selenium-enriched chemotherapeutic effects, making them a valuable anticarcinogenic food crop in the fight against human illnesses [124]. Extracts from cactus pear cladodes have demonstrated anticancer activity against prostate and breast cancer cell lines [125].

Cactus pear cladodes are rich in nutrients such as polyphenols, fatty acids, vitamins, natural pigments, and antioxidants, making them a generally safe choice for human consumption. They are often valued for their potential health benefits, including anti-inflammatory properties, reduced risk of chronic diseases, cancer prevention, and immune function support [126].

While cactus pear cladodes are non-toxic for most individuals, some may experience mild digestive issues, such as diarrhea or stomach cramps, particularly if consumed in large quantities or by those with sensitive digestive systems [126,127]. It is also important to ensure that cactus pear cladodes are properly prepared and cooked. If raw or improperly handled, they may harbor harmful bacteria [126,127,128]. Overall, when consumed in moderation and prepared safely, cactus pear cladodes can be a nutritious addition to a balanced diet.

Cooking and processing methods may significantly influence the bioavailability of bioactive compounds in cactus pear cladodes. Some compounds are heat-sensitive, and cooking methods such as boiling or steaming can lead to the loss of these nutrients, while gentler techniques (e.g., sautéing) may help preserve them. According to De Santiago et al. [129], cooking—except for boiling—increases both soluble and insoluble fiber in cactus cladodes, while heat treatments can enhance antioxidant capacity and phenolic content. Microwaving and griddling are preferred methods for maximizing bioactive compounds; whereas, boiling is associated with a loss of soluble nutrients in cladodes [68].

Mechanical processing techniques, such as chopping, blending, or juicing, can break down cell walls, making bioactive compounds more accessible for absorption, thereby increasing their bioavailability. Fermentation can also enhance the bioavailability of certain compounds by breaking down complex polysaccharides and producing beneficial metabolites such as short-chain fatty acids [130].

Preservation techniques, such as drying or pickling, can affect the stability of bioactive substances. While dried cladodes may preserve their nutrients, this preservation depends on factors such as the harvest time and drying temperature [131]. Therefore, to maximize the bioavailability of bioactive components in cactus pear cladodes, it is crucial to select appropriate cooking, processing, and preservation methods. Using gentler techniques and combining cladodes with complementary foods can further enhance their nutritional value.

### 3.1. Antioxidant Activity

Due to their high content of bioactive phytochemicals, several plant parts and byproducts are used as antioxidants, in functional foods, in traditional medicine, and in the cosmetic industry [132,133,134,135]. Numerous studies have explored the relationship between fruit and/or vegetable-based diets and oxidative-stress-related diseases, such as cardiovascular, cerebrovascular, and cancer, with a particular emphasis on the role of antioxidant molecules in disease prevention. Many plant parts and organs contain bioactive compounds with health-promoting properties. For example, vitamins C and E are known to protect DNA and cell tissue from free radical damage, playing a crucial role in the prevention of cancer, hypertension, and some cardiac disorders [133,136,137].

Cactus pears contain a variety of bioactive substances with antioxidant properties, including polyphenols, natural pigments, and vitamins C and E [138,139,140]. These phytochemicals contribute significantly to the health benefits of cactus pears [138,141]. The antioxidant properties of cactus pear cladodes are influenced by factors such as genotype, maturity stage, and cooking techniques [142,143]. Some cultivars exhibit higher total phenolic and flavonoid contents, resulting in enhanced antioxidant activity. This highlights the importance of selecting specific cultivars to maximize the health benefits associated with the antioxidant properties of cactus cladodes.

Additionally, the antioxidant activity of cactus cladode extracts can be affected by the solvent used for extraction, as observed in other plant species. The solubility of phytochemicals in different solvents impacts extraction efficiency and subsequent antioxidant activity [144,145]. The antioxidant activity of cactus cladode extracts is typically assessed using the 2,2-diphenyl-1-picrylhydrazyl (DPPH) scavenging activity method. The literature indicates that the choice of solvent can significantly influence DPPH radical scavenging activity [117,146].

Cooking techniques, such as griddling and microwaving, may significantly influence the bioavailability of phenolic compounds in cactus pear cladodes. These cooking methods help preserve a greater portion of the antioxidant properties. After digestion, microwave-cooked samples exhibit the highest levels of bioaccessible polyphenols and antioxidant activity [142].

Polyphenols, particularly flavonoids and phenolic acids, have been extensively studied in cactus pear due to their antioxidant properties. These compounds interact with lipids, proteins, and carbohydrates to prevent oxidation, creating an antioxidative environment by either directly scavenging free radicals or interacting with various enzyme systems [139,147]. Thus, a cactus-based diet can benefit human health by reducing oxidative stress, minimizing oxidative damage, and stabilizing free radicals [148,149,150]. According to Butera et al. [149], aqueous extracts of cactus pears exhibit potent antioxidant activity due to bioactive compounds, such as betalains and ascorbic acid, which help reduce lipid oxidation and enhance the body’s redox balance. Cactus pear byproducts, such as cladode juice, have also demonstrated strong antioxidant activity [151].

### 3.2. Antidiabetic and Antiobesity Properties

Cactus cladodes present a promising natural alternative for managing diabetes. This is due to their high fiber content, bioactive molecules, and ability to enhance insulin sensitivity. Cactus cladodes are rich in bioactive compounds, including dietary fiber, polysaccharides, vitamins, and minerals. The high fiber content slows glucose absorption in the intestines, lowers postprandial blood glucose levels, which is critical for type 2 diabetics, and improves blood sugar control [152]. Clinical studies have demonstrated that cactus cladodes can effectively reduce blood sugar levels and improve lipid profiles, providing additional benefits for diabetics who face an increased risk of cardiovascular diseases [153].

Cactus pear cladodes are rich in polyphenols, which play a crucial role in diabetes prevention by modulating key signaling pathways and mechanisms. These compounds scavenge free radicals, reducing oxidative stress that can damage pancreatic β-cells and impair insulin secretion, thereby supporting healthy insulin production. Additionally, polyphenols enhance insulin sensitivity, potentially through the activation of AMP-activated protein kinase (AMPK), promoting glucose uptake and fatty acid oxidation in muscle and adipose tissues. They also inhibit pro-inflammatory cytokines, such as TNF-α and IL-6, and modulate inflammatory pathways, such as NF-κB, thus reducing systemic inflammation—a significant contributor to insulin resistance [154].

Moreover, polyphenols influence enzymes involved in carbohydrate metabolism, such as α-glucosidase and hexokinase, which slow carbohydrate digestion and enhance glucose utilization. They help regulate lipid metabolism by promoting fatty acid oxidation and reducing lipogenesis, often through pathways involving peroxisome proliferator-activated receptors (PPARs). Additionally, polyphenols can affect hormones related to glucose regulation, such as glucagon-like peptide-1 (GLP-1), which enhances insulin secretion and promotes satiety. They also modulate signaling cascades, including the PI3K/Akt pathway, which is crucial for insulin signaling, thus improving glucose uptake in cells. Through these diverse mechanisms, polyphenols contribute to better glucose homeostasis and insulin sensitivity, helping to prevent the onset of diabetes. Their ability to target multiple pathways makes them valuable components in dietary strategies for metabolic health [155,156].

Numerous studies have reported the hypoglycemic effects of different cactus pear species. Frati et al. [157] and Bwititi et al. [158] highlighted the antidiabetic properties of the stems and cladodes of *O. ficus indica* and *O. megacantha*. Nazareno [159] reported that the young cladodes of *O. streptacantha* exhibit a hypoglycemic effect. Yang et al. [160] demonstrated that the polysaccharides found in the cladodes of *O. monacantha* significantly lower blood glucose levels.

Cactus cladodes contain polysaccharides, including mucilage, which may improve insulin sensitivity and glucose uptake in cells [122]. Cactus cladodes provide essential vitamins and minerals that contribute to overall health. Magnesium is associated with a reduced risk of type 2 diabetes due to its role in insulin action and glucose metabolism [161]. Glutamic acid may be involved in insulin synthesis and glucose metabolism [162].

Today, the use of plant extracts and byproducts is emerging as an effective strategy for managing obesity. Numerous studies have shown that consuming cactus pear cladodes and their byproducts can lower blood glucose levels and reduce the risk of obesity [160,163,164]. Uebelhack et al. [163] found that cactus pear fibers promote weight loss by increasing fecal fat excretion, binding to dietary fat, and facilitating its elimination. Aboura [164] noted that the polyphenol content of cactus pears possesses anti-inflammatory properties that may mitigate inflammation associated with obesity and colitis.

Cladode powders can inhibit adipogenesis by reducing triglyceride accumulation and glucose absorption in adipocytes. In vivo studies involving high-fat diet rat models revealed that supplementation with these powders reduced body weight gain, while improving metabolic parameters [165]. Furthermore, bioactive compounds and dietary fibers can help with weight management by enhancing satiety and decreasing calorie intake [166,167]. These fibers also play a role in modulating gut microbiota, which is increasingly recognized for its impact on obesity and metabolic health [168].

### 3.3. Antihyperlipidemic and Anticholesterolemic Activities

Cladodes from *O. ficus indica* exhibit remarkable antihyperlipidemic and anticholesterolemic activities, attributed to their high phytochemical profile and antioxidant properties. These cladodes can improve lipid profiles by lowering total cholesterol and triglyceride levels, which highlights their potential as natural agents for controlling hyperlipidemia through the modulation of lipid metabolism and the enhancement of antioxidant defenses, contributing to cholesterol level reduction [169]. Cactus cladodes have anticholesterolemic properties due to their high fiber content and phytosterols, which help limit cholesterol absorption in the intestines [20]. These characteristics not only contribute to lower blood cholesterol levels but also promote cardiovascular health [170].

Wolfram et al. [171] indicated that non-diabetic and non-obese patients who consumed cactus pears (*O. robusta*) experienced reductions in total cholesterol (12%), low-density lipoprotein cholesterol (15%), apolipoprotein B (9%), triglycerides (12%), fibrinogen (11%), blood glucose (11%), insulin (11%), and uric acid (10%). These benefits can be attributed to the fiber (pectin) content of cactus pear. Padilla-Camberos et al. [172] found that aqueous extracts of cactus pear (*O. ficus indica*) inhibit pancreatic lipase, thereby preventing hypercholesterolemia, a mechanism linked to the polyphenolic content of cactus pears. Wolfram et al. [173] suggested that regular consumption of cactus pears lowers total and LDL cholesterol, while enhancing platelet function.

### 3.4. Antiulcer Activity

Cactus pear cladodes have been used in traditional medicine for centuries to treat gastric ulcers and promote healing [150]. Flavonoids, polysaccharides, phytosterols, and antioxidants in cactus cladodes interact to significantly enhance their antiulcer properties, making them an effective natural therapy for stomach ulcers [174]. The administration of lyophilized cladodes of *O. ficus indica* has been shown to preserve the cytoarchitecture of the gastric mucosa and protect against ethanol-induced ulcers, resulting in substantial antiulcer activity [174].

The flavonoids found in cactus pear cladodes act as antioxidants, reducing oxidative stress in the gastrointestinal mucosa and preventing ulcer development. They also promote stomach tissue repair [175]. Polysaccharides, or complex carbohydrates, protect the gastrointestinal system by enhancing mucosal defense mechanisms and promoting ulcer healing [176]. The phytochemicals found in cactus cladode extracts possess anti-inflammatory properties that protect the stomach lining, reducing inflammation and promoting mucosal integrity. The antioxidants in cactus cladodes neutralize free radicals, preserve the gastrointestinal lining, and help to prevent ulcer formation [177].

### 3.5. Antimicrobial Activity

Many plant species have demonstrated potent antibacterial activity in their extracts [178,179,180]. Cactus cladodes, in particular, exhibit remarkable antibacterial properties against a wide range of bacterial strains, which are attributed to their rich phytochemical composition. The antimicrobial activity of cactus cladode extracts has been tested against various bacteria, including *Escherichia coli*, *Salmonella typhi*, *Helicobacter pylori*, *Staphylococcus aureus*, *Bacillus subtilis*, and *Bacillus cereus* [153,181].

The phytochemical composition of cactus pear cladodes can vary among species, leading to variations in their antimicrobial efficacy. Cactus cladodes contain high levels of gallic acid, a phenolic compound recognized for its antibacterial effects [182,183]. Additionally, cactus pear cladodes contain a variety of other phenolic compounds, which can damage bacterial cell membranes and interfere with metabolic processes, thereby inhibiting bacterial growth. Some saponin compounds found in cactus pear cladodes also exhibit antibacterial properties [184].

Several investigations have demonstrated the antimicrobial properties of cactus extracts. Ennouri et al. [185] showed that hexane extracts of *O. inermis* were effective against *E. coli* and *Staphylococcus aureus*. R’bia et al. [186] found that the antioxidant and antibacterial properties of cactus pear varied significantly depending on the oil fraction used. Their findings revealed that the unsaponifiable fraction exhibited superior biological activity compared to the glyceride fractions. The unsaponifiable fraction has been proven to be more efficient against various pathogenic bacteria, particularly *E. coli*, making it a promising candidate for use as a preservative or food additive.

Extracts from *Opuntia* species, including *O. ficus indica* and *O. dillenii*, have also demonstrated antifungal activity against common fungal infections [15,187]. For example, some extracts were effective against species known to cause human infections, such as *Candida* and *Aspergillus* [188]. Volatile extracts from various parts of three cactus pear species (*O. lindheimeri* var linguiformis, *O. macrorhiza*, and *O. microdasys*) exhibited pronounced antifungal effects against certain phytopathogenic fungi, with leaf extracts showing particularly strong activity. However, these volatile extracts had no effect on *Botrytis cinerea*, *Fusarium solani* f. sp. cucurbitae, and *Pythium ultimum* [189]. The antifungal properties of cactus pear cladodes suggest potential applications in food preservation, alternative therapies for fungal infections, and cosmetic products targeting fungal-related skin conditions [190].

Ahmad et al. [191] reported that *O. streptacantha* cladode extracts possess antiviral properties, demonstrating the ability to suppress DNA and RNA virus replication, including equine herpes virus, pseudorabies virus, influenza type A virus, respiratory syncytial virus, and human immunodeficiency virus (HIV-1). Nazareno [159] also found that cactus pears have antiviral effects against herpes simplex and influenza A viruses, with chlorophyll derivatives serving as the active components.

### 3.6. Anti-Inflammatory and Analgesic Effects

Several investigations have demonstrated that extracts from *O. ficus indica* fruits, cladodes, and stems possess strong anti-inflammatory effects due to their bioactive compounds [192,193,194]. Cactus cladodes are particularly rich in anti-inflammatory phytochemicals, including flavonoids and phenolic compounds [195]. Cladodes of *O. ficus indica* significantly reduced the production of chemokine (IL-8 and TNF-α) and nitric oxide (NO) in human intestinal Caco-2/TC7 cells, resulting in anti-inflammatory activity [130].

The major components in *Opuntia* species responsible for anti-inflammatory activity are phenolic compounds, particularly catechin, chlorogenic acid, ferulic acid, and P-coumaric acid [195]. Catechin is a strong antioxidant that mitigates oxidative stress and inflammation [196]. Chlorogenic acid may inhibit pro-inflammatory cytokines [197], while ferulic acid contributes to health-promoting effects [115]. P-coumaric acid has also been shown to reduce inflammation [198].

Lactic acid fermentation of *Opuntia* cladodes produces flavonoid derivatives with enhanced antioxidant and anti-inflammatory properties. Fermented cladode pulp has been found to inhibit IL-8, TNFα, and PGE2 production compared to unfermented controls [130]. The primary flavonoid responsible for anti-inflammatory effects is quercetin, which inhibits the production of pro-inflammatory mediators [199]. Kaempferol, another flavonoid, also possesses anti-inflammatory properties and regulates the immune response [200]. Isorhamnetin, an antioxidant, may help reduce inflammation [201].

Many other components of cactus pear cladodes may also contribute to their anti-inflammatory properties. For example, tocopherols, including α-, β-, and γ-tocopherols, act as antioxidants that reduce oxidative stress linked to inflammation [202]. Gallic acid, found in cactus pear cladodes, is recognized for its anti-inflammatory effects [203].

Recent research suggests that cactus pear cladodes may also possess centrally mediated analgesic effects. According to Park et al. [204], ethanolic extracts from *O. ficus indica* inhibited acetic-acid-induced writhing syndrome, suggesting an analgesic effect. The aqueous extracts of *O. ficus indica* have also shown analgesic properties. In fact, based on two thermal stimulus methods (the tail flick and hot plate tests), aqueous extracts of cactus pear cladodes showed a significant increase in latency time at doses of 300, 500, and 1000 mg/kg body weight [205]. Moreover, the methanolic extract of *O. ficus indica* effectively alleviated pain in animal models, particularly in hot plate tests, where various doses of the extract reduced pain responses, with 40 mg/kg providing significant analgesic benefits [206].

### 3.7. Anticancer Effect

The potential use of cactus pear cladodes as a natural source of anticancer compounds has been investigated. The bioactive compounds of cactus pear cladodes exhibited lethal effects on cancer cells while enhancing overall cellular defense through antioxidant mechanisms. Cactus pear cladodes demonstrated anticancer properties in several ways, including inducing programmed cancer cell death, which is crucial for effective cancer treatment, reducing oxidative stress, protecting DNA, and promoting cellular health and longevity. Furthermore, it has been shown that cactus cladode extracts can protect against radiation-induced DNA damage, suggesting their potential use in conjunction with radiotherapy [184,207].

Cactus pear cladodes are rich in polyphenols, which play a vital role in cancer prevention through various mechanisms that modulate key signaling pathways. By scavenging free radicals, polyphenols reduce oxidative stress—a factor linked to DNA damage and cancer development—thereby helping to maintain cellular integrity. Additionally, they inhibit pro-inflammatory signaling pathways, such as NF-κB and COX-2, since chronic inflammation is associated with cancer progression [208]. By mitigating inflammation, polyphenols may help prevent tumor growth [154].

Polyphenols can induce programmed cell death (apoptosis) in cancer cells by activating caspase pathways and inhibiting antiapoptotic proteins, such as Bcl-2, which increases cancer cell mortality. They also influence cell cycle regulation by modulating cyclins and cyclin-dependent kinases (CDKs). Compounds such as resveratrol can induce cell cycle arrest, thereby preventing cancer cell proliferation. Moreover, polyphenols affect transcription factors, such as Nrf2 and P53, which regulate genes involved in antioxidant defense, cell cycle control, and apoptosis, thereby enhancing cellular responses to stress and inhibiting tumor development. Some polyphenols also interact with hormone receptors, influencing pathways related to hormone-dependent cancers, such as breast and prostate cancers, by modulating estrogen signaling and potentially reducing the risk of estrogen-driven tumors. Through these multifaceted mechanisms, polyphenols emerge as a significant focus in dietary and therapeutic strategies for cancer prevention [154,208,209].

Zou et al. [210] found that the aqueous extract of cactus pears can inhibit the proliferation of cervical, ovarian, and breast cancer cell lines. This growth suppression was associated with an increase in apoptotic cells and cell cycle arrest in the G1 phase. In another study, Chavez-Santoscoy et al. [211] investigated the effects of cactus pear juice on the vitality of four cancer cell lines—mammary, prostate, colon, and hepatic—compared to regular fibroblast. Their findings revealed that the viability of prostate and colon cancer cells was most significantly affected. The findings of this study revealed that *O. violaceae* has a high flavonoid concentration, which reduces the viability of prostate and colon cancer cells, while sparing breast or liver cancer cells. On the other hand, juice from *O. rastrera* reduced the proliferation of all four cancer cell lines without adversely affecting the viability of normal fibroblasts.

## 4. Conclusions and Future Perspectives

The cactus pear is a plant genus widely used in the food, cosmetic, and pharmaceutical industries. This is mainly due to the bioactive compounds found in its various parts, including cladodes. While most studies have focused on the bioactive compounds found in cactus pear fruits, cladodes have received comparatively less attention from scientists. The present review outlined the structure–function relationship of the main nutraceuticals found in cactus pear cladodes and presented their concentration ranges, as reported in the literature, taking into account species and geographical origin.

Today, cactus pear cladodes are not only a staple in the traditional cuisines of several countries but also offer a wide range of applications in the food, health, and industrial sectors. Their nutritional benefits have attracted significant attention in various commercial markets. The commercial applications of cactus pear cladodes are diverse and promising. As consumer preferences increasingly lean toward healthier, sustainable, and natural products, the potential for cactus pear cladodes in various markets continues to expand.

Harnessing these applications can foster both economic growth and environmental sustainability, making cactus pear cladodes a valuable resource for the future. They can be prepared in numerous ways—grilled, boiled, or sautéed—and are commonly featured in salads, tacos, and stews across many American countries. Their mild flavor and unique texture appeal to health-conscious consumers seeking low-calorie, high-fiber options.

Additionally, the richness of cactus pear cladodes in vitamins, minerals, and antioxidants has led to a rising popularity in dietary supplements, which are available in powder, capsule, and extract forms, marketed for benefits such as blood sugar regulation, weight management, and overall wellness. Cactus pear cladodes are also increasingly included in a variety of beverages, with their hydrating properties and refreshing taste making them an attractive addition to functional drinks aimed at health-oriented consumers. The growing trend of plant-based and functional beverages further enhances the market potential of cactus pear cladodes.

In the cosmetic industry, the moisturizing and soothing properties of cactus pear cladodes have led to their incorporation in skincare products. Ongoing and emerging research is exploring the pharmacological properties of cactus pear cladodes, paving the way for innovative medicinal products.

Most studies on the nutraceutical composition and applications of cactus pear cladodes have focused on *O. ficus indica*, the most economically important species. More research is needed to assess the nutraceutical content of other cactus pear species that have received limited attention. Exploring these species could unlock new opportunities for targeted and sustainable utilization, as well as optimize the industrial applications of cactus pear cladodes.

Despite the data presented in this review on the bioactive compounds of cactus pear cladodes, some limitations should be acknowledged. The lack of human clinical studies highlights significant limitations in our understanding of the health benefits associated with cactus pear cladodes. Although many studies suggest that cactus pear cladodes are rich in antioxidants, fatty acids, vitamins, pigments, and other bioactive compounds, the absence of robust clinical trials hampers us from drawing definitive conclusions about their efficacy and optimal consumption methods. Additionally, further research is needed to explore the bioavailability of these compounds, particularly how cooking and processing affect them. It is also essential to investigate the specific molecular pathways and signaling processes that contribute to the health benefits of cactus pear cladodes. Individual variations in metabolism and digestion can influence the absorption and utilization of these compounds in the body. This gap in knowledge underscores the necessity for more comprehensive studies to evaluate both the availability and potential therapeutic effects of cactus pears, ultimately paving the way for evidence-based dietary recommendations.

Other challenges and limitations associated with the use of cactus pear cladodes include their cost, availability, and potential allergenic properties. Cultivating and harvesting cactus pears can be resource-intensive, requiring specific conditions and careful management. Additionally, costs may vary by region and demand, making it less economically viable for some producers. Pest susceptibility can further impact yields and sustainability. For example, the spread of the cochineal scale insect (*Dactylopius opuntiae*) in some regions and countries has complicated cactus pear cultivation and increased costs. Moreover, the limited growing season for cladodes can affect the consistency of supply. Individuals with allergies to specific fruits or vegetables may also react adversely to cladodes, underscoring the need for caution.

## Figures and Tables

**Table 1:** Examples of nutraceuticals present in cactus pear cladodes based on species, cultivar, and geographic origin.

Species	Cultivar/Genotype	Geographic Origin	Polyphenols	Fatty Acids			Vitamins		Amino Acids	Natural Pigments		Phytosterols	Reference
			Flavonoids	Linolenic Acid	Linoleic Acid	Oleic Acid	Vitamin C/ Ascorbic Acid	Vitamin E/Tocopherols	Glutamic Acid	Betacyanins + Betaxanthins	β-Carotene		
*O. ficus indica*	Copena F1	Mexico	18.1 mg QE/g DW	-	-	-	-	-	-	-	-	-	[37]
*O. ficus indica*	Jalpa	Mexico	36.6 mg QE/g DW	-	-	-	-	-	-	-	-	-	[37]
*O. ficus indica*	Copena V1	Mexico	16.0 mg QE/g DW	-	-	-	-	-	-	-	-	-	[37]
*O. ficus indica*	Villanueva	Mexico	24.7 mg QE/g DW	-	-	-	-	-	-	-	-	-	[37]
*O. ficus indica*	Real de Catorce	Mexico	15.4 mg QE/g DW	-	-	-	-	-	-	-	-	-	[37]
*O. ficus indica*	Cristalino	Mexico	22.5 mg QE/g DW	-	-	-	-	-	-	-	-	-	[37]
*O. ficus indica*	Forrajero Mina	Mexico	18.9 mg QE/g DW	-	-	-	-	-	-	-	-	-	[37]
*O. streptacantha*	Cardon Blanco	Mexico	17.9 mg QE/g DW	-	-	-	-	-	-	-	-	-	[37]
*O. ficus indica*	Gymno-Carpo	South Africa	-	-	-	-	77.40 mg/100 g ascorbic acid	-	-	5.40 mg/kg	-	-	[38]
*O. ficus indica*	Meyers	South Africa	-	-	-	-	67.25 mg/100 g ascorbic acid	-	-	12.82 mg/kg	-	-	[38]
*O. ficus indica*	Nepgen	South Africa	-	-	-	-	26.78 mg/100 g ascorbic acid	-	-	8.07 mg/kg	-	-	[38]
*O. ficus indica*	Ofer	South Africa	-	-	-	-	58.74 mg/100 g ascorbic acid	-	-	20.15 mg/kg	-	-	[38]
*O. robusta*	Robusta	South Africa	-	-	-	-	24.04 mg/100 g ascorbic acid	-	-	29.20 mg/kg	-	-	[38]
*O. ficus indica*	Milpa Alta	Mexico	11.2–18.6 mg CE/g	-	-	-	-	-	-	-	-	18.7–33.2 mM equivalents/g	[113]
*O. ficus indica*	-	Egypt	-	20.19%	24.81%	10.39%	-	-	-	-	57.51 μg/100 g	-	[18]
*O. ficus indica*	-	South Korea	1.29 mg/g	-	-	-	71.2 mg% vitamin C	-	1543.15 mg%	-	-	-	[39]
*O. ficus indica*	-	Ethiopia	6.44–25 mg CE/g DW	-	-	-	-	-	-	-	-	-	[41]
*O. ficus indica*	NT	Spain	-	8.85–16.4%	12.8–34.7%	8.52–16.3%	-	-	-	-	-	-	[54]
*O. ficus indica*	NO	Spain	-	15.9–20.4%	27.9–33.9%	9.22–14.2%	-	-	-	-	-	-	[54]
*O. ficus indica*	NE	Spain	-	5.31–16.3%	20.3–37.6%	10.7–23.7%	-	-	-	-	-	-	[54]
*O. ficus indica*	NA	Spain	-	11.5–19.7%	19.8–27.7%	15.0–21.1%	-	-	-	-	-	-	[54]
*O. ficus indica*	FR	Spain	-	1.69–8.68%	16.3–53.8%	22.3–24.8%	-	-	-	-	-	-	[54]
*O. ficus indica*	NJ	Spain	-	10.5–13.4%	25.1–25.8%	21.6–36.3%	-	-	-	-	-	-	[54]
-	Blanco sin Espinas	Mexico	-	-	-	-	-	-	-	-	26.0–32.5 µg/g FW	-	[100]
-	Blanco con Espinas	Mexico	-	-	-	-	-	-	-	-	14.5–16.0 µg/g FW	-	[100]
-	Verde Valtierrilla	Mexico	-	-	-	-	-	-	-	-	26.0–32.5 µg/g FW	-	[100]
*O. ficus indica*	-	Mexico	-	-	-	-	-	-	-	-	76–119 µg/g dry basis	-	[101]
*O. ficus indica*	Milpa Alta	Mexico	-	-	-	-	▪ 63.92 mg/100 g dry matter total vitamin C ▪ 19.21 mg/100 g dry matter ascorbic acid	-	-	-	61.32 mg/100 g dry matter	-	[68]
*O. ficus indica*	Atlixco	Mexico	-	-	-	-	▪ 57.03 mg/100 g dry matter total vitamin C ▪ 25.52 mg/100 g dry matter ascorbic acid	-	-	-	66.85 mg/100 g dry matter	-	[68]
*O. ficus indica*	Redonda	Mexico	-	-	-	-	-	-	1.29–2.22 g/100 g of protein	-	-	-	[84]
*O. humifusa*	-	South Korea	-	17.20%	38.88%	8.42%	17.33 mg% vitamin C	-	609.90 mg/100 g	-	-	-	[55]
*O. microdasys*	-	Tunisia	-	12.2%	20%	5.7%	0.0061 g/100 g DW ascorbic acid	6.9 mg/100 g DW total tocopherols	-	-	-	-	[57]
*O. macrorhiza*	-	Tunisia	-	10.9%	24%	5.9%	0.017 g/100 g DW ascorbic acid	5.1 mg/100 g DW total tocopherols	-	-	-	-	[57]
*O. Sulphurea*	-	Argentina	-	27.41%	25.58%	11.40%	-	-	-	-	-	-	[56]

The symbol ‘-’ means data not available.
